# Effects of Light-at-Night on the Rat Liver – A Role for the Autonomic Nervous System

**DOI:** 10.3389/fnins.2019.00647

**Published:** 2019-06-20

**Authors:** Anne-Loes Opperhuizen, Ewout Foppen, Martijs Jonker, Paul Wackers, Martijn van Faassen, Michel van Weeghel, Linda van Kerkhof, Eric Fliers, Andries Kalsbeek

**Affiliations:** ^1^Hypothalamic Integration Mechanisms, Netherlands Institute for Neuroscience, Amsterdam, Netherlands; ^2^Laboratory of Endocrinology, Amsterdam University Medical Center, Department of Clinical Chemistry, University of Amsterdam, Amsterdam, Netherlands; ^3^MAD – Dutch Genomics Service and Support Provider, Swammerdam Institute for Life Sciences, University of Amsterdam, Amsterdam, Netherlands; ^4^Centre for Health Protection, National Institute for Public Health and the Environment, Bilthoven, Netherlands; ^5^Department of Laboratory Medicine, University Medical Centre Groningen, University of Groningen, Groningen, Netherlands; ^6^Laboratory Genetic Metabolic Diseases, Amsterdam University Medical Center, University of Amsterdam, Amsterdam, Netherlands; ^7^Amsterdam University Medical Center, Department of Endocrinology and Metabolism, University of Amsterdam, Amsterdam, Netherlands

**Keywords:** transcriptome, metabolome, biological clock, neural pathway, circadian

## Abstract

Exposure to light at night (LAN) has been associated with serious pathologies, including obesity, diabetes and cancer. Recently we showed that 2 h of LAN impaired glucose tolerance in rats. Several studies have suggested that the autonomic nervous system (ANS) plays an important role in communicating these acute effects of LAN to the periphery. Here, we investigated the acute effects of LAN on the liver transcriptome of male Wistar rats. Expression levels of individual genes were not markedly affected by LAN, nevertheless pathway analysis revealed clustered changes in a number of endocrine pathways. Subsequently, we used selective hepatic denervations [sympathetic (Sx), parasympathetic (Px), total (Tx, i.e., Sx plus Px), sham] to investigate the involvement of the ANS in the effects observed. Surgical removal of the sympathetic or parasympathetic hepatic branches of the ANS resulted in many, but small changes in the liver transcriptome, including a pathway involved with circadian clock regulation, but it clearly separated the four denervation groups. On the other hand, analysis of the liver metabolome was not able to separate the denervation groups, and only 6 out of 78 metabolites were significantly up- or downregulated after denervations. Finally, removal of the sympathetic and parasympathetic hepatic nerves combined with LAN exposure clearly modulated the effects of LAN on the liver transcriptome, but left most endocrine pathways unaffected.

**Conclusion:** One-hour light-at-night acutely affects the liver transcriptome. Part of this effect is mediated via the nervous innervation, as a hepatectomy modulated and reduced the effect of LAN on liver transcripts.

## Introduction

Nowadays, artificial light competes with the importance of sunlight for determining the timing of human daily activities. Light exposure at night (LAN) is increasingly affecting organisms worldwide (Falchi et al., 2016) and associated with numerous pathologies in humans, including metabolic disorders such as obesity and type 2 diabetes mellitus (Obayashi et al., 2013; McFadden et al., 2014). Human and animal studies have reported adverse effects of chronic and acute LAN exposure on release of hormones including melatonin (Kalsbeek et al., 1999; Kozaki et al., 2016; Albreiki et al., 2017) and cortisol/corticosterone (Ishida et al., 2005), as well as on sleep and arousal (Daneault et al., 2016; Pilorz et al., 2016) and on glucose metabolism (Fonken et al., 2010; Coomans et al., 2013; Cheung et al., 2016; Albreiki et al., 2017; Opperhuizen et al., 2017; Versteeg et al., 2017). Previously, our lab showed that 2 h of LAN acutely decreased glucose tolerance in rats. LAN exposure at the beginning of the dark phase increased glucose concentrations during an intravenous glucose tolerance test without changing insulin levels (Opperhuizen et al., 2017). One of the underlying mechanisms might be light-induced stimulation of glucose production by the liver. Indeed, in 2009 we showed that 1 h of LAN exposure caused an upregulation of *phosphoenolpyruvate carboxykinase*, an important enzyme for glucose production, mRNA expression in the rat liver (Cailotto et al., 2009). Together these data suggest that the liver is important for the effects of artificial light at night on glucose tolerance.

Previous studies in rodents have shown that nocturnal light exposure acutely affects the activity of the autonomic nervous system (ANS), involving both sympathetic and parasympathetic branches innervating the liver, as well as those to a number of other organs (Niijima et al., 1992, 1993; Mutoh et al., 2003; Ishida et al., 2005). The involvement of the ANS in the effects of nocturnal light was further shown by the fact that removal of the autonomic innervation abolished light-induced changes in the liver (Cailotto et al., 2009) and the adrenal (Ishida et al., 2005). Therefore, we hypothesized that the liver is an important target organ for the effects of LAN on glucose tolerance as reported previously (Opperhuizen et al., 2017) and that these effects may be (partly) executed via the ANS. Of course, in addition to these ANS mediated effects, the effects of LAN on the liver may also involve hormonal and behavioral pathways, as these effects are not mutually exclusive.

The autonomic innervation of the liver is well known to be involved in the control of glucose and lipid metabolism (Shimazu and Fukuda, 1965; Bruinstroop et al., 2013; Bisschop et al., 2015; Mizuno and Ueno, 2017; Martinez-Sanchez et al., 2017). In general, the sympathetic branch is dominant when the organism requires an active state, whereas the parasympathetic branch is leading during rest. A balance between the often complementary sympathetic and parasympathetic branches of the ANS is required to result in the appropriate physiological control (Buijs, 2013). The activity of both branches is controlled by pre-autonomic neurons found, amongst others, in the paraventricular nucleus of the hypothalamus (La Fleur et al., 2000; Buijs et al., 2003; Buijs, 2013). These neurons were identified in studies using injections of viral trans-synaptic tracers in peripheral organs including liver, adipose tissue, adrenal glands, and pancreas (Buijs et al., 1999; La Fleur et al., 2000; Buijs et al., 2001; Kreier et al., 2002). Light information from the retina may reach these pre-autonomic neurons via its projections to the biological clock in the suprachiasmatic nucleus.

Disruption of autonomic hepatic innervation has been associated with metabolic pathologies. Patients receiving liver transplants frequently suffer from metabolic derangements as well as immune system failure (Luca et al., 2015; Russo, 2017; Thoefner et al., 2018), likely due to the consequential (partial) disruption of autonomic input to the liver. Moreover, metabolic diseases, such as non-alcoholic fatty liver disease, obesity and type 2 diabetes mellitus, include symptoms of autonomic deregulation, sometimes even manifested prior to disease development (Licht et al., 2013; Wulsin et al., 2015).

The primary goal of the present study was to investigate: (1) the acute effects of exposure to light at night on the liver transcriptome and (2) the possible involvement of the ANS in the effects observed. We investigated this in male rats using selective hepatic denervation of the ANS. To optimize the denervation conditions we studied the liver metabolome and transcriptome after different hepatic denervations in a pilot experiment that preceded the main LAN plus denervation experiment.

## Materials and Methods

### Animals and Housing

Male Wistar rats (Charles River Breeding Laboratories, Sulzfeld, Germany) weighing 280–300 g were housed in constant conditions with a controlled 12:12/light:dark cycle (lights on at 7:00, lights off at 19:00), constant temperature (21 ± 2°C), humidity (60 ± 5%) and background noise (radio). Animals had *ad libitum* access to water and regular chow (Teklad Global Diet, Harlan, Horst, the Netherlands) during the complete experiment, unless stated otherwise. All experimental procedures performed were in accordance with the Council Directive 2010/63EU of the European Parliament and the Council of 22 September 2010 on protection of animals used for scientific purposes. All experimental procedures performed were approved by the Animal Ethics Committee of the Royal Dutch Academy of Arts and Sciences (KNAW, Amsterdam) and were in accordance to the guidelines on animal experimentation of the Netherlands Institute for Neuroscience (NIN).

### Pilot Experiment

In order to determine the best experimental condition for the liver denervation we first performed a pilot experiment in which animals were randomly assigned to one of five surgical groups, 1 week after adaptation to the animal facility:

**Intact** (*n* = 5): Animals were not operated upon.
**Sham** (*n* = 5): Animals received a sham hepatic denervation.
**Sympathectomy (Sx)** (*n* = 7): Animals received a sympathetic hepatic denervation.
**Parasympathectomy (Px)** (*n* = 7): Animals received a parasympathetic hepatic denervation.
**Total hepatectomy (Tx)** (*n* = 7): Animals received a sympathetic plus parasympathetic hepatic denervation.

#### Surgery

Animals in all groups were anesthetized with 80 mg/kg ketamine (Eurovet Animal Health, Bladel, Netherlands) and 8 mg/kg Rompun (xylaxine, Bayer Health Care, Mijdrecht, Netherlands). Intact animals only received anaesthesia and were left to recover. Sham surgery included the laparotomy and destruction of connective tissue (i.e., breaking of membranes beneath the skin and disruption of small blood vessels surrounding the autonomic nerves) but did not include destruction of any parasympathetic or sympathetic nerve branches to the liver. Denervations were performed as previously reported (Kalsbeek et al., 2004).

The hepatic sympathectomy initiated with finding the hepatic portal vein where the hepatic artery breaks up into hepatic artery proper and gastroduodenal artery. Blunt dissection separated the arteries from the portal vein. Nerve bundles that run along the hepatic artery proper were removed with the use of a microscope. Attached connective tissue was also removed between hepatic artery and portal vein to further eliminate nerve crossings.

During the parasympathectomy surgery, moving the stomach and esophagus exposes the fascia containing the hepatic branch. The hepatic branch separates from the vagal trunk and this was cut between ventral vagus trunk and liver. In addition, smaller branches between stomach and liver were transected.

Total denervation (Tx) included removal of both sympathetic and parasympathetic branches to the liver. The same, very experienced, experimenter (EFo) performed all surgeries. After surgery, animals were monitored in an incubator (30°C) until wakening. Afterward animals were returned to their home cage and left to recover in group-housing and monitored closely.

#### Experimental Design

At the experimental day, 2 weeks after surgery, food was removed 4 h prior to sacrifice. Animals were anesthetized with O_2_:CO_2_ (20%:80%) gas for 1 min before decapitation at 13:00 (i.e., 6h after lights on). Immediately the abdominal cavity was opened and a ∼200 mg piece of the left lateral lobe of the liver was obtained and snap frozen in liquid nitrogen. All tissues were stored in −80°C until further processing. Tissue collection of 10–11 animals was finished within 1 h. Collection of tissues of all animals (*n* = 31) was spread over 3 days to minimize variation in the daily timing of sacrifice. The same experimenter (ALO) obtained all liver samples. We excluded animals with incomplete recovery (i.e., insufficient recovery of bodyweight or wound healing) or incomplete denervation (i.e., noradrenaline content >10% for Sx- or Tx-animals; based on our previous experiments all Px-denervations were assumed to be successful; Kalsbeek et al., 2004). Thus, two animals from the Sx-, Px- and Tx-groups and one animal from the Intact-group (total *n* = 7) were excluded and twenty-four samples (*n* = 4/5/5/5/5 from groups Intact/Sham/Sx/Px/Tx, respectively) were analyzed.

### Liver Denervation and Light at Night (LAN)

Animals were randomly assigned to one of three experimental groups, 1 week after adaptation to the animal facility:

**Sham-DARK** (*n* = 8): Animals received a sham hepatic denervation. At the experimental day, animals remained in darkness and were sacrificed at 22:00 (i.e., 3 h after lights off).
**Sham-LAN** (*n* = 8): Animals received a sham hepatic denervation. At the experimental day, animals were exposed to light at night from 21:00 to 22:00 and sacrificed at 22:00.
**Tx-LAN** (*n* = 10): Animals received a total hepatic denervation. At the experimental day, animals were exposed to light at night from 21:00 to 22:00 and sacrificed at 22:00.

Surgeries were done by the methods described in the pilot experiment. The same experimenter (EFo) performed all surgeries. After surgery, animals were monitored in an incubator (30°C) until wakening and afterward returned to their home cage and left to recover in-group housing. After 1 week, animals were housed individually for another week and monitored closely until sacrifice.

#### Experimental Design

At the experimental day, 2 weeks after surgery, food was removed at 21:00 until sacrifice (22:00). Light exposure was done with mixed white TL-light from the ceiling of ∼125 ± 25 photopic lux and animals remained in their own cage and were not touched or moved during the exposure. After the light exposure or darkness (Sham-DARK), all animals were sacrificed by the method described in Experiment 1. Tissue collection of 13 animals was finished within 1 h, and collection of all tissues from all animals (*n* = 26) was spread and randomized over 2 days to minimize variation in the daily timing of sacrifice. The same experimenter (ALO) obtained all liver samples. Two animals from Tx-LAN were excluded due to incomplete recovery (i.e., insufficient body weight recovery) and incomplete denervation (i.e., noradrenaline content >10%). In total, 24 samples (*n* = 8/group) were analyzed by microarray analysis.

### RNA Isolation

The following steps were similar for tissues from both experiments. Liver tissue was cut on dry ice with a razor blade (max. 5 mm), added to 1 mL of TRIzol (Macherey-Nagel Ltd., Oensingen, Switzerland) and homogenized by Ultra Turrax homogenizer (IKA, Staufen, Germany). Total RNA was isolated from tissues using TRIzol reagent (Macherey-Nagel Ltd., Oensingen, Switzerland). RNA concentration was determined using NanoDrop DS (Thermo Fisher Scientific, Wilmington, DE, United States) and RNA quality was determined using BioAnalyzer (Agilent, Santa Clara, CA, United States). Ten microliter of RNA was used for microarray analysis. Samples included in the microarray analysis had RNA concentration >100 ng/μl and RIN values for RNA quality >8.0.

### Noradrenaline

Liver tissue was cut on dry ice, added to 100 mg tissue/mL homogenous solution (0.08 M acetic acid + 80 mg/mL glutathione) and homogenized by Ultra Turrax homogenizer (IKA, Staufen, Germany). Samples were centrifuged (4°C, 2,500 × *g*, 15 min) and supernatant was stored at −80°C. Noradrenaline concentration was determined by LC-MS/MS as described previously (van Vliet et al., 2015).

### Microarray Procedure

Amplification of 100 ng total RNA was done using the GeneChip 3′ IVT Plus Reagent kit (Thermo Fisher Scientific, Waltham, MA, United States) generating biotinylated complementary RNA. The labeled samples were hybridized to GeneChip HT RG-230 PM arrays (Thermo Fisher Scientific). GeneTitan Wash and Stain Kit for 3′ IVT Array Plates were used to perform washing and staining steps. Scanning was performed with the GeneTitan Instrument (Thermo Fisher Scientific).

### Metabolomics Procedure

Two mg of freeze-dried liver tissue of the pilot experiment was used for metabolomics analysis (Koenis et al., 2018). A Thermo Fisher Scientific ultra-high pressure liquid chromatography system (Waltham, MA, United States) coupled to Thermo Q Exactive (Plus) Orbitrap mass spectrometer (Waltham, MA, United States) was used. Two mg of freeze dried liver tissue was transferred in a 2 mL tube and the following internal standards, dissolved in MQ, were added, D_3_-aspartic acid, D_3_-serine, D_5_-glutamine, D_3_-glutamate, ^13^C_3_-pyruvate, ^13^C_6_-isoleucine, ^13^C_6_-glucose, ^13^C_6_-fructose-1,6-biphosphate, ^13^C_6_-glucose-6-phosphate, adenosine-^15^N_5_-monophosphate and guanosine-^15^N_5_-monophosphate (5 μM). MQ was added to a total volume of 500 μL and subsequently 500 μL of MeOH and 1 mL of chloroform was added. Samples were kept on ice, vortexed, sonicated by needle sonication for 30 s at 5 W output and centrifuged for 10 min at 14.000 rpm at 4°C. The “polar” top layer ∼800 μL was transferred to a new 1.5 mL tube and dried to dryness in a vacuum concentrator. Dried samples were dissolved in 100 μL methanol/water (6/4; v/v). For the analysis, we used a Thermo Fisher Scientific ultra-high pressure liquid chromatography system (Waltham, MA, United States) coupled to Thermo Q Exactive (Plus) Orbitrap mass spectrometer (Waltham, MA, United States). The autosampler was held at 10°C during the runs and 5 μL of sample was injected on the analytical column. The chromatographic separation was established using a SeQuant ZIC-cHILIC column (PEEK 100 × 2.1 mm, 3.0 μm particle size, Merck, Darmstadt, Germany) and kept at 15°C. The flow rate was 0.250 mL/min. The mobile phase was composed of (A) 9/1 acetonitrile/water with 5 mM ammonium acetate; pH 6.8 and (B) 1/9 acetonitrile/water with 5 mM ammonium acetate; pH 6.8, respectively. The LC gradient program was: beginning with 100% (A) hold 0–3 min; ramping 3–20 min to 36% (A); ramping from 20 to 24 min to 20% (A); hold from 24 to 27 min at 20% (A); ramping from 27 to 28 min to 100% (A); and re-equilibrate from 28 to 35 min with 100% (A). The MS data were acquired in negative ionization mode at full scan range at 140,000 resolution.

### Statistical Analysis

#### Microarray Data

The raw data per experiment were subjected to a set of quality controls. The quality checks revealed no significant hybridization and experimental blocking effects. All arrays used in the final analysis passed the quality control and were annotated (Carlson, 2015; Project, 2015). Normalized expression values were calculated using the robust multi-chip average (RMA) algorithm (Irizarry et al., 2003). Differential gene expression between the experimental groups and the control group were statistically analyzed using the Limma package (Phipson et al., 2017) in R-3.2.1^1^. False Discovery Rate corrected *p*-values (*q*-values) were calculated according to Storey et al. (2015). The top 5% genes according to their *q*-value were mapped against the KEGG database (Tenenbaum, 2017) for pathway analysis. *P*-values were calculated using a statistical test based on the hypergeometric distribution. Venn diagrams of differentially expressed genes (DEGs) were created of top 100 DEGs (based on *p*-value) for the pilot experiment and with DEGs *p* < 0.001 for the LAN experiment. Venn diagrams of significant pathways for both experiments contain pathways with *p* < 0.05 (similar to Schaap et al., 2012). Heatmaps were created with the top 500 DEGs (based on *p*-value) for Sham vs. Intact comparison and top 100 DEGs (based on *p*-value) of included comparisons: Sham vs. Px, Sham vs. Sx and Sham vs. Tx for the pilot experiment and Sham-DARK vs. Sham-LAN, Sham-LAN vs. Tx-LAN and Sham-DARK vs. Tx-LAN for the LAN experiment. Data are clustered on genes and samples. Heatmaps of the LAN experiment include horizontal representation of the cluster analysis dividing the heatmap in 7 clusters. The clusters were further analyzed by pathway analysis as described above.

Since the animals from the pilot experiment and LAN experiment were sacrificed at different times of the day, i.e., 6 h after lights on for the pilot experiment and 3 h after lights off for the LAN experiment, the microarray data from the 2 experiments cannot be compared directly.

#### Metabolomics Data

Interpretation of the metabolomics data was performed in the Xcalibur software (Thermo Fisher Scientific, Walthman, MA, United States). The amount of metabolite is a ratio based on the internal standards used. Statistical analysis and the generation of the heatmap and volcano plot was performed using the programming language R.

#### Body Weight and Noradrenaline

All data are expressed as mean ± standard error of the mean (SEM). Body weight and noradrenaline were analyzed with One-way ANOVA to test for group effects. If a significant effect was found, *post-hoc* Tuckey’s multiple comparisons test was performed. Results were considered significant when *p* < 0.05.

## Results

### Pilot Experiment

**FIGURE 1:**
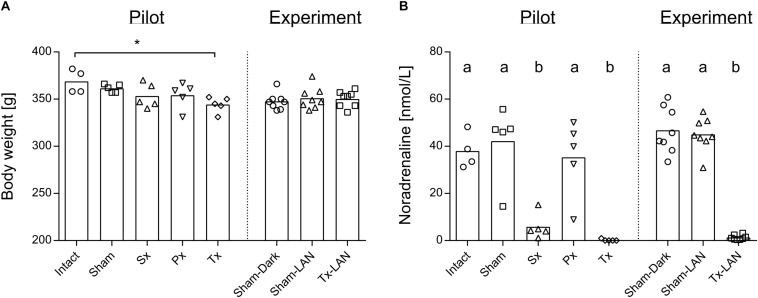
Body weight and noradrenaline content of the liver of experimental animals. **(A)** Body weight at sacrifice of rats was slightly reduced in Tx-animals when compared to Intact in the pilot Experiment. Level of significance ^*^p < 0.05. Body weight was equal between all groups in the LAN Experiment. **(B)** Noradrenaline content of liver at sacrifice is reduced in animals that received a sympathectomy or total denervation, both in the pilot and LAN experiment. Level of significance ab; *p* < 0.05.

Removal of the sympathetic (Sx), parasympathetic (Px) or both nerves (Tx) did not significantly affect body weight, when compared to the two control groups, i.e., receiving laparotomy (Sham) or anaesthesia only (Intact). Body weight after recovery was nearly equal between all groups, except for a small difference between Intact- and Tx-animals (*p* = 0.03; Figure 1A). Noradrenaline content in liver tissue was assessed in all groups to confirm whether removal of the sympathetic nerve was successful in Sx- and Tx-animals (<10% of control values). Significantly reduced levels of noradrenaline were found in Sx- and Tx-animals compared to animals with intact sympathetic nerves (*p* < 0.0001, Figure 1B), indicating successful removal of the sympathetic nerve in Sx- and Tx-animals. One Sx-animal (Sx4 in A) had a relatively high noradrenaline content. One Px-animal and one Sham-animal (Px5 and Sham4 respectively in Figure 4A) showed relatively low noradrenaline content (Figure 1B).

**FIGURE 2:**
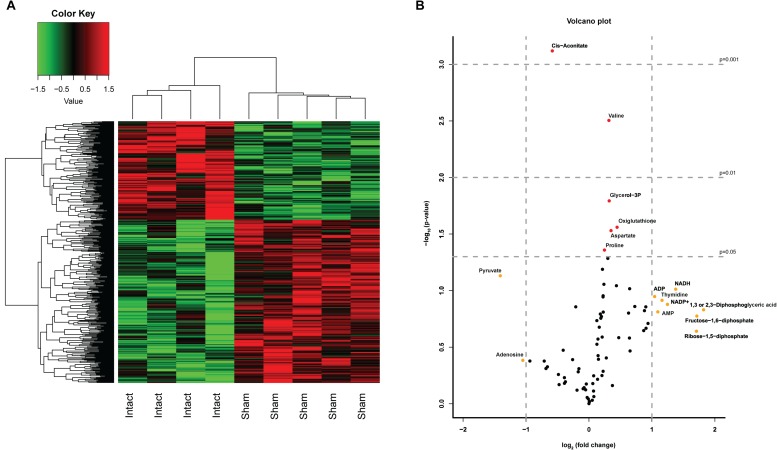
Effects of a Sham surgery on liver transcriptome and metabolome. **(A)** Heatmap of top 500 DEGs between Sham and Intact animals. A laparotomy caused a clearly distinct expression pattern even after 2 weeks of recovery. **(B)** Volcano Plot depicting the analysis of 78 metabolites in Sham-denervated and Intact-animals. The volcano plot shows a large number of metabolites with a divergent expression pattern due to the Sham surgery. Metabolites identified in red showed statistical significant changes of *p* < 0.05 (−log10 > 1.3) *p* < 0.01 (−log10 > 2) or *p* < 0.001 (−log10 > 3), metabolites in yellow showed a fold change > 2 (log2FC > 1).

**FIGURE 3:**
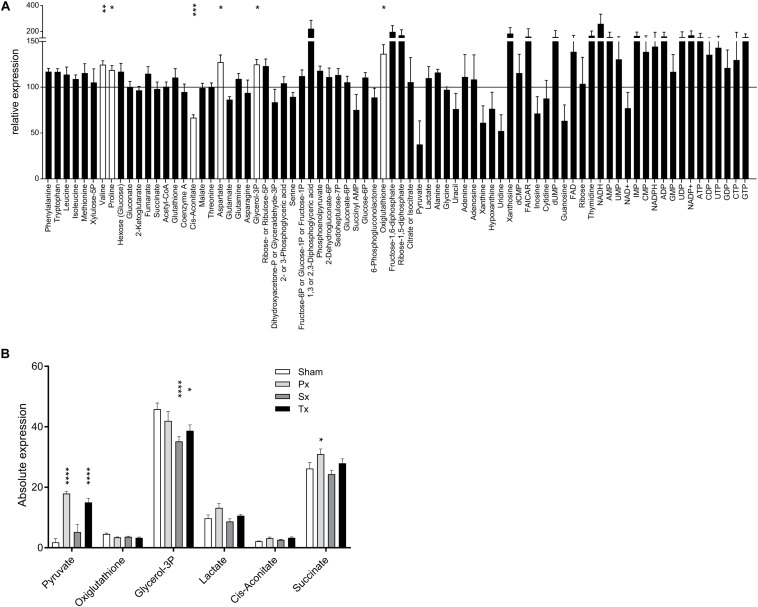
Liver metabolome after liver denervation. **(A)** Seventy-eight metabolites are depicted comparing Sham-denervated and Intact-animals. Six metabolites were significantly affected by Sham-surgery (white bars). Data are normalized to Intact-expression (i.e., 100%). *t*-test ^*^p < 0.05; ^∗∗^*p* < 0.01; ^∗∗∗^*p* < 0.001; ^****^p < 0.0001. **(B)** Absolute expression of the 6 metabolites is shown that were significantly up- and downregulated due to sympathetic, parasympathetic or total liver denervation.

In order to investigate whether both control groups, i.e., non-operated and sham-operated, were necessary to control for the effect of the laparotomy, we first compared the results of the Intact- and Sham-animals. We studied the effect of a laparotomy by microarray analysis (>30,000 transcripts) and a semi-targeted metabolomics approach on 78 metabolites. The heatmap of the microarray analysis clearly shows the inequality of the two control groups (Figure 2A) with 1 DEG with *q* < 0.05 and 34 DEGs (*p* < 0.001) affected by the laparotomy. Analysis of the 78 metabolites revealed a less pronounced separation of the two control groups. Nevertheless, volcano plot analysis (Figure 2B) revealed 10 metabolites with a fold change (FC) > 2 (log2(FC) > 1 or < -1, depicted in yellow) and 6 metabolites with a significant change *p* < 0.05 [-10log(pval) > 1.3, depicted in red], including one with a significant change *p* < 0.01 and one with a change *p* < 0.001 due to the Sham-surgery. The laparotomy significantly increased the expression of proline, aspartate, glycerol-3phosphate, oxiglutathione (*p* < 0.05) and valine (*p* < 0.01), whereas it decreased cis-aconitate (*p* < 0.001; Figure 3A). From these metabolomic and transcriptomic data, we concluded that the laparotomy induces slightly discernible effects on the liver metabolome and transcriptome, despite a recovery time of approximately 2 weeks. Therefore, for the remainder of the analyses on the effects of hepatic denervations we removed the Intact-animals group, in order to better compare within-experiment effects between surgical groups.

Clustering-based heatmap analysis (data not shown) of the metabolomics data revealed no clustering of treatment groups. This indicates that there are no global metabolite changes due to the hepatectomies, which is in contrast to the transcriptomics data (discussed in the next section). However, analysis of individual metabolites showed that selective or total denervation of the liver did induce significant changes (as compared to sham-surgery) in some of the liver metabolites. Multiple comparison analysis revealed significant up- or downregulation of six metabolites (Figure 3B). *Post-hoc* analysis revealed that pyruvate was significantly upregulated in Px- and Tx-animals compared to Sham. Px-denervation induced significant upregulation of succinate when compared to Sham-animals. Sx- and Tx-animals showed significantly reduced glycerol-3P when compared to Sham.

**FIGURE 4:**
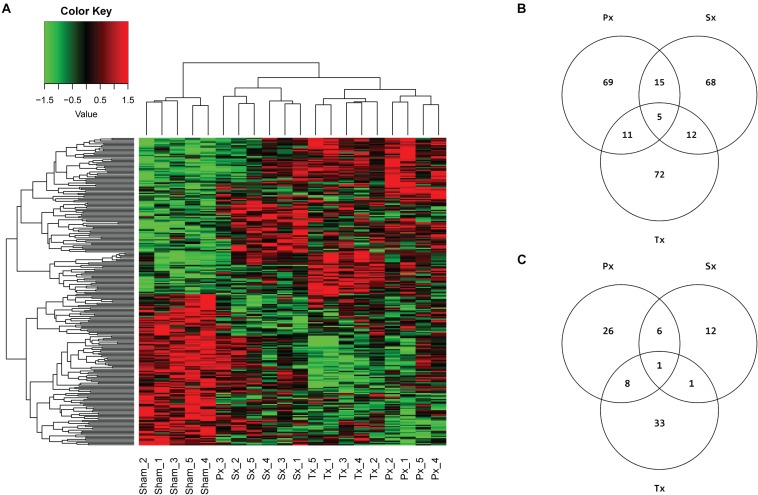
Microarray analysis of hepatic denervations. **(A)** Heatmap, clustered on samples and genes, with 252 unique genes resulting from top 100 DEGs of 3 comparisons (Sham vs. Px, Sham vs. Sx, Sham vs. Tx), based on *p*-value. Individual animals are presented in columns. Biological groups separate completely, except for sample Px3, which is aligned between Sham- and Sx-animals. **(B)** Venn diagram of top 100 DEGs per comparison (Px vs. Sham, Sx vs. Sham, Tx vs. Sham) and the number of overlapping genes between comparisons. **(C)** Venn diagram showing the number of pathways that are significantly (*p* < 0.05) enriched with top 5% (FDR corrected) DEGs by Sx-, Px- or Tx-denervation when compared to Sham-animals. Tx-denervation resulted in most pathways significantly enriched. One pathway was overlapping between all non-Sham denervation groups, and one other pathway overlapped between Sx- and Tx-animals. Most overlapping pathways were found between Px- and Tx-groups (9 overlapping).

Microarray analysis of ∼30,000 transcripts in liver tissue of Sx-, Px-, Tx-, and Sham-animals was performed to identify specific differentially expressed genes (DEGs) and/or pathways uniquely affected by removal of the sympathetic (Sx) or parasympathetic (Px) branch of the ANS. A total denervation by removal of both nerves (Tx) induced significant (*q* < 0.05) differential expression of 17 transcripts (Supplementary Table 1). However, Sx or Px of the liver did not cause any single gene to be significantly (*q* < 0.05) up- or downregulated. Analysis of the top 100 DEGs (based on *p*-value) between the Sham-group and each denervation-group (Sx vs. Sham, Px vs. Sham, and Tx vs. Sham, i.e., 3 comparisons) resulted in 252 affected genes (Figure 4A). Of these 252 genes 69, 68, and 72 genes were uniquely affected by Px, Sx, and Tx, respectively (Figure 4B). The heatmap shows nearly complete separation of the four biological groups, except for one sample in the Px-group, which aligned between Sham- and Sx- animals (Figure 4A). This sample (Px3) may be divergent from the others because the animal needed a short extra isoflurane anesthesia to re-stitch the wound at the first day after surgery.

Pathway analysis is helpful to identify whether the observed, subtle, changes in gene expression are related to particular biological functions. The top 5% of DEGs (based on *p*-value, FDR corrected) was used to study pathway enrichment. Out of 301 possible pathways, Sx-animals showed significant enrichment of 20 pathways when comparing to Sham, whereas Px- and Tx-surgeries induced enrichment of 41 and 43 pathways (Figure 4C and Supplementary Tables 2–4), respectively. Nine pathways overlapped between Px and Tx, and only 2 pathways overlapped between Sx and Tx (Figure 4C and Table 1). One pathway was affected in all denervation groups when compared to Sham: *AGE-RAGE signaling pathway in diabetic complications* (Table 1).

**TABLE 1:** List of pathways significantly affected by a liver denervation.

**Pathway name**	**Number**	**n genes**	**Overlap in Px**	**% affected**	***p*-value Px**	**Overlap in Tx**	**% affected**	***p*-value Tx**			
**Overlapping pathways between Px- and Tx-animals**											
Circadian rhythm	04710	30	7	23.3	0.0006	6	20.0	0.0033			
Colorectal cancer	05210	64	10	15.6	0.0012	7	10.9	0.0401			
Thyroid hormone signaling pathway	04919	119	13	10.9	0.0066	11	9.2	0.0362			
Acute myeloid leukemia	05221	57	8	14.0	0.0072	8	14.0	0.0072			
Adherens junction	04520	74	8	10.8	0.0312	9	12.2	0.0113			
Protein processing in endoplasmic reticulum	04141	165	14	8.5	0.0376	22	13.3	0.0000			
Protein export	03060	26	4	15.4	0.0387	4	15.4	0.0387			
FoxO signaling pathway	04068	136	12	8.8	0.0402	22	16.2	0.0000			
**Overlapping pathways between Sx- and Tx-animals**											
ErbB signaling pathway	04012	91	10	11.0	0.0155	9	9.9	0.0382			

**Pathway name**	**Number**	**n genes**	**Overlap in Px**	**% affected**	***p*-value Px**	**Overlap in Sx**	**% affected**	***p*-value Sx**	**Overlap in Tx**	**% affected**	***p*-value Tx**

**Overlapping pathways between Sx-, Px-, and Tx-animals**											
**AGE-RAGE signaling pathway in diabetic complications**	04933	104	16	15.4	0.0001	12	11.5	0.0058	11	10.6	0.01493

**Common pathways significantly (p < 0.05) enriched with top 5% (FDR corrected) DEGs in both Px- and Tx-animals (upper part), in Sx- and Tx-animals (middle part) and between Sx-, Px-, and Tx-animals (low part), when compared to Sham animals. Sorted on significance. n genes the number of genes in the pathway; overlap the number of genes of the pathway present in list of DEGs; % affected the overlap between the genes in the pathway and the genes in list of DEGs expressed as a percentage; p-value the level of significance in enrichment.**

Based on the results of this pilot experiment, we made the following choices for the LAN experiment:

(1) Only include experimental groups with abdominal (sham-) surgery, i.e., not to include a non-operated control group in order to better compare within-experiment effects between surgical groups;
(2) Only include a total-denervation group, since this was the only denervation group showing significant changes in gene expression; and
(3) Only perform a microarray analysis since using the metabolomics data we were not able to separate the different experimental groups.

### Liver Denervation and Light at Night (LAN)

Three experimental groups were included to study whether 1 h acute exposure to light at night (LAN) induces direct changes in the liver transcriptome and whether this is mediated through the autonomic nervous system: Sham-animals sacrificed in the dark (Sham-DARK), Sham-animals sacrificed after LAN (Sham-LAN) and Tx-denervated animals sacrificed after LAN (Tx-LAN). Thus, all animals received abdominal (sham) surgery. Body weight was unaffected by the procedures (Figure 1A). Noradrenaline content of the liver was significantly reduced by removal of the sympathetic nerve in Tx-animals (*p* < 0.0001, Figure 1B).

**FIGURE 5:**
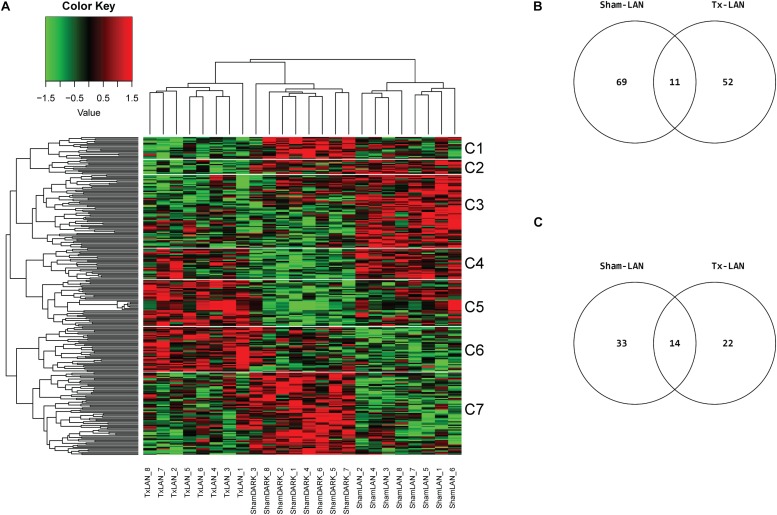
Microarray analysis of LAN exposure with and without hepatic denervation. **(A)** Heatmap of top 100 DEGs of 3 comparisons (Sham-DARK vs. Sham-LAN, Sham-LAN vs. Tx-LAN, Sham-DARK vs. Tx-LAN), presents expression profile of 244 unique genes. Individual subjects (animals) are presented by columns. Data are clustered on genes and samples. White lines and C-numbers on the right indicate clustered groups of genes responsible for the differences between biological groups. **(B)** Venn representing the number of affected DEGs (*p* < 0.001) when comparing Sham-LAN with Sham-DARK and Tx-LAN with Sham-DARK. Eleven DEGs were affected by both Sham-LAN and Tx-LAN when compared to Sham-DARK. **(C)** Venn diagram representing the number of pathways significantly (*p* < 0.05) enriched with top 5% of genes, based on *p*-value (FDR corrected), after exposure to LAN in Sham-animals compared to Sham-DARK animals and Tx-animals compared to Sham-DARK animals.

None of the genes investigated was significantly (*q* < 0.05) up- or downregulated by LAN. Selection of the top 100 DEGs based on *p*-value (*p* < 0.05) resulted in 244 unique DEGs of the three possible comparisons (i.e., Sham-LAN vs. Sham-DARK, Tx-LAN vs. Sham-DARK, and Sham-LAN vs. Tx-LAN) after analysis of all transcripts. Clustering on genes and samples completely separated the three biological groups, as all samples aligned within their experimental group (Figure 5A). Comparing Sham-DARK with Sham-LAN animals resulted in 80 DEGs (*p* < 0.001) and 47 pathways (*p* < 0.05, Supplementary Table 5) affected by LAN (Figures 5B,C). Several of these 47 pathways turned out to be involved in endocrine signaling, including *oxytocin signaling, bile secretion, estrogen signaling, thyroid signaling* and *glucagon signaling*. Exposure to LAN after Tx-denervation reduced the number of DEGs (*n* = 63; *p* < 0.001) and pathways (*n* = 36; *p* < 0.05) affected when compared to Sham-DARK (Figures 5B,C and Supplementary Table 6 for common pathways Sham-LAN and Tx-LAN).

The heatmap divides in seven clusters of genes that were affected by either of the interventions. Pathway analysis on genes within these seven separate clusters (Table 2) shows a number of pathways accounting for the discrepancy between biological groups. Clusters 1 and 7 contain genes that were downregulated by LAN, both in the presence or absence of hepatic nerves. These genes are related to a number of pathway involved in immune functions, amongst others. Genes in clusters 4 and 5 were upregulated by LAN in both the Sham and Tx-group. Genes in cluster 3 were upregulated by LAN, but this effect was prevented by the total denervation. The genes in this cluster are mainly involved in bile acid biosynthesis and drugs metabolism. Cluster 6 contains genes that were not affected by LAN in the Sham-animals, but upregulated by LAN when the autonomic nerves had been removed. The opposite was seen in cluster 2, as these genes were not affected by LAN in the Sham-group, but were downregulated in the denervated group.

**TABLE 2:** Significantly enriched pathways found in cluster analysis of LAN experiment.

**Pathway name**	**Number**	**Ngenes**	**Overlap**	***P*-value**
**CLUSTER 1**				
TNF signaling pathway	04668	110	2	0.0021
Focal adhesion	04510	206	2	0.0070
Selenocompound metabolism	00450	19	1	0.0116
Cocaine addiction	05030	47	1	0.0284
Cytosolic DNA-sensing pathway	04623	56	1	0.0337
Colorectal cancer	05210	64	1	0.0385
Amphetamine addiction	05031	65	1	0.0391
Inflammatory bowel disease (IBD)	05321	65	1	0.0391
Renal cell carcinoma	05211	68	1	0.0408
Pertussis	05133	73	1	0.0438
B cell receptor signaling pathway	04662	74	1	0.0444
Leishmaniasis	05140	75	1	0.0449
Bacterial invasion of epithelial cells	05100	81	1	0.0484
Salmonella infection	05132	83	1	0.0496
**CLUSTER 2**				
Cysteine and methionine metabolism	00270	47	1	0.0195
**CLUSTER 3**				
Metabolism of xenobiotics by cytochrome P450	00980	70	4	0.0000
Chemical carcinogenesis	05204	91	3	0.0009
Glutathione metabolism	00480	59	2	0.0064
Drug metabolism - cytochrome P450	00982	71	2	0.0092
Bile secretion	04976	72	2	0.0095
Platinum drug resistance	01524	82	2	0.0121
Taurine and hypotaurine metabolism	00430	11	1	0.0221
Ubiquitin mediated proteolysis	04120	141	2	0.0334
Glycosaminoglycan biosynthesis - heparan sulfate / heparin	00534	25	1	0.0495
**CLUSTER 4**				
Primary bile acid biosynthesis	00120	16	1	0.0138
**CLUSTER 5**				
Glycine. serine and threonine metabolism	00260	40	2	0.0012
African trypanosomiasis	05143	40	2	0.0012
Malaria	05144	59	2	0.0026
Purine metabolism	00230	182	2	0.0231
Dorso-ventral axis formation	04320	26	1	0.0330
**CLUSTER 6**				
Ras signaling pathway	04014	232	3	0.0031
Influenza A	05164	171	2	0.0196
alpha-Linolenic acid metabolism	00592	25	1	0.0310
MAPK signaling pathway	04010	259	2	0.0420
African trypanosomiasis	05143	40	1	0.0491
**CLUSTER 7**				
Influenza A	05164	171	5	0.0000
Fc gamma R-mediated phagocytosis	04666	91	4	0.0000
Epstein-Barr virus infection	05169	231	5	0.0002
Viral carcinogenesis	05203	239	5	0.0002
Measles	05162	138	4	0.0003
Leukocyte transendothelial migration	04670	120	3	0.0027
Osteoclast differentiation	04380	134	3	0.0036
Hepatitis B	05161	139	3	0.0040
Adrenergic signaling in cardiomyocytes	04261	148	3	0.0048
Protein processing in endoplasmic reticulum	04141	165	3	0.0065
Longevity regulating pathway - multiple species	04213	65	2	0.0098
Fc epsilon RI signaling pathway	04664	70	2	0.0113
Inositol phosphate metabolism	00562	74	2	0.0125
Chronic myeloid leukemia	05220	76	2	0.0132
Herpes simplex infection	05168	220	3	0.0141
Regulation of actin cytoskeleton	04810	221	3	0.0143
Phosphatidylinositol signaling system	04070	96	2	0.0205
Estrogen signaling pathway	04915	96	2	0.0205
Natural killer cell mediated cytotoxicity	04650	102	2	0.0229
Amoebiasis	05146	106	2	0.0246
Oocyte meiosis	04114	113	2	0.0277
Toxoplasmosis	05145	123	2	0.0324
Sphingolipid signaling pathway	04071	124	2	0.0329
Cell cycle	04110	127	2	0.0344
Hepatitis C	05160	129	2	0.0353
Glycosaminoglycan biosynthesis - keratan sulfate	00533	16	1	0.0360
Apoptosis	04210	141	2	0.0415
PI3K-Akt signaling pathway	04151	336	3	0.0419
Phospholipase D signaling pathway	04072	148	2	0.0453
Glycosaminoglycan biosynthesis - chondroitin sulfate / dermatan sulfate	00532	21	1	0.0470
Hippo signaling pathway	04390	156	2	0.0498

**The microarray heatmap of LAN Experiment was divided in 7 clusters of DEGs showing similar response to intervention (LAN or Tx-LAN).**

## Discussion

The present study clearly shows that exposure to artificial light at night (LAN) acutely affects the liver transcriptome. Moreover, removal of the autonomic nervous input to the liver changed the effects of LAN on the liver transcriptome, but did not completely abolish its effects, indicating that in addition to the ANS LAN signals also use other pathways to affect the liver transcriptome.

The hypothalamus has long been appreciated to be pivotal in the control and coordination of homeostatic activity. Historically, this has been viewed in terms of the extensive neuroendocrine control system resulting from processing of hypothalamic signals relayed to the pituitary. Through these actions, endocrine signals are integrated throughout the body, modulating a vast array of physiological processes (Bruinstroop et al., 2014). More recently, the control emanating from the hypothalamus over the ANS has been increasingly recognized as a powerful additional modulator of peripheral tissues. The neural regulation is believed to be fine, selective and rapid, whereas the hormonal regulation is more stable and widespread.

Nocturnal light exposure has clear physiological effects, particularly via synchronization of the circadian system. Depending on its timing, nocturnal light exposure will either advance or delay the phase of the endogenous circadian pacemaker. These phase-shifting effects of nocturnal light are mediated via a direct projection from the retina to the central pacemaker in the brain located in the suprachiasmatic nuclei (SCN) in the anterior hypothalamus. Subsequently the timing information from the SCN is distributed to the rest of the brain and body via pre-autonomic and endocrine motor neurons in the nearby hypothalamus (Buijs and Kalsbeek, 2001). In addition to these well-known circadian effects, nocturnal light exposure also has direct physiological effects by changing locomotor activity, body temperature (Scheer et al., 2005) and hormone release (Niijima et al., 1992, 1993; Buijs et al., 1999; Mutoh et al., 2003; Ishida et al., 2005; Kiessling et al., 2014). Far less is known about the neural pathways involved in these direct effects, although lesion and denervation experiments have indicated the involvement of both the SCN and the ANS (La Fleur et al., 2000; Buijs et al., 2003; Buijs, 2013).

The current experiment provides further evidence for the involvement of the ANS in the direct effects of LAN. Exposure to LAN caused the differential expression (*p* < 0.001) of >200 hepatic genes. Removal of the autonomic input to the liver clearly modulated the effects of LAN. A large group of upregulated genes by LAN was not affected anymore after the hepatic denervation (cluster #3, *n* = 63). On the other hand, two smaller groups of genes were only affected by LAN after the hepatic denervation (clusters #2 and #6, respectively, *n* = 13 and *n* = 39). Moreover, the number of pathways affected by LAN was reduced from 47 to 36 after hepatic denervation. Finally, the denervated-LAN-exposed animals resembled more the expression pattern of unexposed animals than that of the LAN-exposed animals, an additional indication that the ANS is an important pathway for light signals to reach the liver.

We earlier observed that 1 h LAN increases hepatic PEPCK expression (Cailotto et al., 2009) and 2 h LAN induces glucose intolerance (Opperhuizen et al., 2017). In the current study we used 1 h LAN exposure in order to investigate the “physiological condition” at the time of the start of the glucose tolerance test in our previous study, i.e., halfway the 2 h light exposure. We expected this might provide an explanation of why glucose tolerance was impaired. We hypothesized that this occurs through (ANS-dependent) stimulation of glucose production (Opperhuizen et al., 2017). This hypothesis was not confirmed nor denied by the current data as we did not find a significant change in PEPCK expression or other genes or pathways directly related to glucoregulatory mechanisms, apart from significant enrichment of the *glucagon signaling* pathway. The absence of a significant effect on PEPCK (and other genes) may be due to the lower sensitivity of the microarray as compared to qPCR. Modulation of the molecular circadian hepatic clock is another potential cellular mechanism of action. Light exposure has been described to affect clock gene expression within a few hours in rat liver (Cailotto et al., 2009) and mouse adrenal gland (Ishida et al., 2005; Kiessling et al., 2014), and this depended on an intact autonomic innervation. Furthermore, direct effects of autonomic activity on the hepatic molecular clock have been described (Terazono et al., 2003; Shibata, 2004). Our current study supports these findings: the *circadian rhythm* pathway was one of the most affected pathways by both a parasympathetic and a total denervation (Table 1 and Supplementary Tables 3, 4). Approximately 20% of the genes in this pathway were modulated by the denervations, including *Cryptochrome 2* and *RAR-related orphan receptor beta* in Tx-animals and *Brain and muscle ARNT-like 1, Cryptochrome 1, Rorα* and *neuronal PAS domain protein 2* in Px-animals. Modifications in core clock gene expression, hypothetically, lead to subsequent effects in clock controlled genes, which are important in a wide spread array of functions including glucose and energy metabolism. Follow-up studies should include detailed analyses of gene expression and circadian behavioral traits. Importantly, although we have longstanding experience in hepatectomies, as yet, there is no non-invasive tool to monitor the success rate of the parasympathetic denervation, which is an important limitation of our study.

In addition to the prominent involvement of the ANS in the effects of LAN observed, the current experiment also provided clear evidence for the involvement of hormonal pathways, including thyroid hormone, bile acids, glucagon, estrogen, gonadotropin-releasing hormone and oxytocin. Most of these endocrine-related pathways were still significantly affected by LAN after liver denervation, except for glucagon and gonadotropin-releasing hormone signaling. Thyroid and bile acid signaling were significantly enriched in Tx-LAN, whereas estrogen and oxytocin signaling were close to significance (*p* = 0.05 and *p* = 0.06, respectively). Most of these pathways are also well-known for their glucoregulatory effects. However, besides the well-described effects of LAN on melatonin and corticosterone only very few studies, in chickens and related to growth responses, have reported associations between light exposure and gonadotropin-releasing hormone (Zhang et al., 2017) and thyroid hormone levels (Yang et al., 2016). Future studies should investigate whether light indeed also affects the hypothalamic-pituitary-gonadal- and hypothalamic-pituitary-thyroid-axis in rodents. In conclusion, 1 h of light exposure induces widespread changes at the level of the hepatic transcriptome, especially involving pathways involved in endocrine signaling. However, in addition a clear involvement of the ANS was revealed, as a part of the effects of LAN were prevented by interrupting the autonomic nervous innervation of the liver. Removal of the autonomic input especially affected the hepatic molecular clock, potentially an efficient method by which the central biological clock and light affect peripheral metabolism. Thus the deleterious effects of disturbed autonomic nervous activity in the development of metabolic diseases, potentially involves a disruption of the local molecular clock system.

## Data Availability

The datasets generated for this study can be found in the public repository Gene Expression Omnibus, accession number: GSE120980.

## Ethics Statement

All experimental procedures performed were in accordance with the Council Directive 2010/63EU of the European Parliament and the Council of 22 September 2010 on protection of animals used for scientific purposes. All experimental procedures performed were approved by the Animal Ethics Committee of the Royal Dutch Academy of Arts and Sciences (KNAW, Amsterdam) and were in accordance to the guidelines on animal experimentation of the Netherlands Institute for Neuroscience (NIN).

## Author Contributions

All authors provided substantial contributions to the conception and design of experiments (A-LO and AK), animal experimentation (A-LO and EF), microarray analysis (MJ and PW), noradrenaline measurements (MvF), metabolomics analysis (MvW), interpretation of data (A-LO, LvK, EFl, and AK), writing (A-LO, EFl, and AK), and revising (A-LO, EF, MJ, PW, MvF, MvW, LvK, EFl, and AK) of the manuscript.

## Conflict of Interest Statement

The authors declare that the research was conducted in the absence of any commercial or financial relationships that could be construed as a potential conflict of interest.

## Footnotes

1 http://cran.r-project.org
