# Supplementary material for: Effects of Light-at-Night on the Rat Liver – A Role for the Autonomic Nervous System
Source: Front Neurosci. 2019 Jun 20;13:647. doi: 10.3389/fnins.2019.00647 (PMC6596368; doi:10.3389/fnins.2019.00647)
Supplement: Supplementary file 1 [file Data_Sheet_1.docx]

**SUPPLEMENTARY DATA**

**Supplementary Table 1.** Total denervation of liver significantly (*q*<0.05) affected 17 transcripts when compared to Sham-animals. *logFC indicates log Fold Change, AveExpr is the average expression of the transcript by Tx- and Sham-animals, t indicates FC divided by standard error*

| Gene IDs | Gene symbols | Gene names | logFC | Ave Expr | t | *p*-value | adj.*p*-val | *q*-values |
| --- | --- | --- | --- | --- | --- | --- | --- | --- |
| 1367553_PM_x_at | Hbb | hemoglobin. beta | 1.0322 | 10.6457 | 7.8127 | <0.0000 | 0.0024 | 0.0021 |
| 1388608_PM_x_at | NA | NA | 0.9084 | 11.2070 | 7.2434 | <0.0000 | 0.0041 | 0.0035 |
| 1371102_PM_x_at | NA | NA | 1.1283 | 9.7896 | 6.9357 | <0.0000 | 0.0054 | 0.0047 |
| 1370239_PM_at | NA | NA | 0.7720 | 11.6522 | 6.6317 | <0.0000 | 0.0066 | 0.0057 |
| 1370240_PM_x_at | NA | NA | 0.7555 | 11.6818 | 6.5132 | <0.0000 | 0.0066 | 0.0057 |
| 1371245_PM_a_at | NA | NA | 1.0557 | 9.4248 | 6.4977 | <0.0000 | 0.0066 | 0.0057 |
| 1387154_PM_at | NA | NA | 0.9042 | 4.8752 | 6.4357 | <0.0000 | 0.0066 | 0.0057 |
| 1375519_PM_at | LOC287167 | globin. alpha | 0.8833 | 7.2684 | 6.4169 | <0.0000 | 0.0066 | 0.0057 |
| 1398496_PM_at | NA | NA | 0.4584 | 4.1810 | 5.8454 | <0.0000 | 0.0225 | 0.0194 |
| 1383889_PM_at | NA | NA | 0.5382 | 4.8419 | 5.6934 | <0.0000 | 0.0290 | 0.0251 |
| 1379368_PM_at | Bcl6 | B-cell CLL/lymphoma 6 | -3.3739 | 6.7638 | -5.4537 | <0.0000 | 0.0432 | 0.0373 |
| 1385986_PM_at | NA | NA | 1.0360 | 4.4672 | 5.4521 | <0.0000 | 0.0432 | 0.0373 |
| 1380306_PM_at | NA | NA | -1.5330 | 3.8529 | -5.4022 | <0.0000 | 0.0450 | 0.0388 |
| 1382314_PM_at | Isg15 | ISG15 ubiquitin-like modifier | 0.9671 | 6.2372 | 5.2894 | <0.0000 | 0.0549 | 0.0474 |
| 1385429_PM_at | NA | NA | -0.5537 | 4.4794 | -5.2348 | <0.0000 | 0.0557 | 0.0481 |
| 1373416_PM_at | Fndc3b | fibronectin type III domain containing 3B | -0.6018 | 7.7515 | -5.2053 | <0.0000 | 0.0557 | 0.0481 |
| 1374117_PM_at | Baiap2 | BAI1-associated protein 2 | -0.6306 | 5.1373 | -5.2030 | <0.0000 | 0.0557 | 0.0481 |

**Supplementary Table 2.** Pathways significantly (*p*<0.05) enriched with DEGs (*p*<0.001) after comparing sympathectomised animals (Sx) with Sham-denervated animals (Sham). *Pathway rno* indicates the pathway number for *rattus norvegicus;* *n genes* the number of genes in the pathway; o*verlap* the number of genes of the pathway present in list of DEGs; *% affected* the overlap between the genes in the pathway and the genes in list of DEGs expressed as a percentage; *p-value* the level of significance in enrichment.

| pathway | pathway rno | n genes | overlap | % affected | *p*-value |
| --- | --- | --- | --- | --- | --- |
| **Hypertrophic cardiomyopathy (HCM)** | 05410 | 85 | 16 | 18.8 | 0.0000 |
| **Dilated cardiomyopathy** | 05414 | 91 | 15 | 16.5 | 0.0000 |
| **Focal adhesion** | 04510 | 206 | 22 | 10.7 | 0.0007 |
| **Protein digestion and absorption** | 04974 | 93 | 12 | 12.9 | 0.0023 |
| **Pathways in cancer** | 05200 | 400 | 33 | 8.3 | 0.0036 |
| **AGE-RAGE signaling pathway in diabetic complications** | 04933 | 104 | 12 | 11.5 | 0.0058 |
| **Proteoglycans in cancer** | 05205 | 208 | 19 | 9.1 | 0.0085 |
| **ECM-receptor interaction** | 04512 | 84 | 10 | 11.9 | 0.0091 |
| **Arrhythmogenic right ventricular cardiomyopathy (ARVC)** | 05412 | 74 | 9 | 12.2 | 0.0113 |
| **African trypanosomiasis** | 05143 | 40 | 6 | 15.0 | 0.0138 |
| **ErbB signaling pathway** | 04012 | 91 | 10 | 11.0 | 0.0155 |
| **Axon guidance** | 04360 | 179 | 16 | 8.9 | 0.0180 |
| **Rap1 signaling pathway** | 04015 | 216 | 18 | 8.3 | 0.0240 |
| **HTLV-I infection** | 05166 | 295 | 23 | 7.8 | 0.0241 |
| **Platelet activation** | 04611 | 127 | 12 | 9.4 | 0.0255 |
| **Transcriptional misregulation in cancer** | 05202 | 176 | 15 | 8.5 | 0.0312 |
| **Amoebiasis** | 05146 | 106 | 10 | 9.4 | 0.0395 |
| **Morphine addiction** | 05032 | 92 | 9 | 9.8 | 0.0405 |
| **Glycine, serine and threonine metabolism** | 00260 | 40 | 5 | 12.5 | 0.0479 |
| **Cardiac muscle contraction** | 04260 | 81 | 8 | 9.9 | 0.0493 |

**Supplementary Table 3.** Pathways significantly (*p*<0.05) enriched with DEGs (*p*<0.001) after comparing parasympathectomised animals (Px) with Sham-denervated animals (Sham). *Pathway rno* indicates the pathway number for *rattus norvegicus;* *n genes* the number of genes in the pathway; o*verlap* the number of genes of the pathway present in list of DEGs; *% affected* the overlap between the genes in the pathway and the genes in list of DEGs expressed as a percentage; *p-value* the level of significance in enrichment.

| pathway | pathway rno | n genes | overlap | % affected | *p*-value |
| --- | --- | --- | --- | --- | --- |
| **AGE-RAGE signalling pathway in diabetic complications** | 04933 | 104 | 16 | 15.4 | 0.0001 |
| **Circadian rhythm** | 04710 | 30 | 7 | 23.3 | 0.0006 |
| **Colorectal cancer** | 05210 | 64 | 10 | 15.6 | 0.0012 |
| **Osteoclast differentiation** | 04380 | 134 | 15 | 11.2 | 0.0029 |
| **Toll-like receptor signalling pathway** | 04620 | 97 | 12 | 12.4 | 0.0033 |
| **Hepatitis C** | 05160 | 129 | 14 | 10.9 | 0.0052 |
| **Thyroid hormone signalling pathway** | 04919 | 119 | 13 | 10.9 | 0.0066 |
| **Acute myeloid leukaemia** | 05221 | 57 | 8 | 14.0 | 0.0072 |
| **Endocrine resistance** | 01522 | 95 | 11 | 15.6 | 0.0078 |
| **Fructose and mannose metabolism** | 00051 | 38 | 6 | 15.8 | 0.0108 |
| **Pathways in cancer** | 05200 | 400 | 31 | 7.8 | 0.0110 |
| **cGMP-PKG signalling pathway** | 04022 | 171 | 16 | 9.4 | 0.0121 |
| **Rap1 signalling pathway** | 04015 | 216 | 19 | 8.8 | 0.0124 |
| **Tight junction** | 04530 | 143 | 14 | 9.8 | 0.0126 |
| **Central carbon metabolism in cancer** | 05230 | 65 | 8 | 12.3 | 0.0154 |
| **HIF-1 signalling pathway** | 04066 | 109 | 11 | 10.1 | 0.0206 |
| **Carbohydrate digestion and absorption** | 04973 | 44 | 6 | 13.6 | 0.0215 |
| **Oestrogen signalling pathway** | 04915 | 96 | 10 | 10.4 | 0.0217 |
| **Apoptosis** | 04210 | 141 | 13 | 9.2 | 0.0247 |
| **Melanoma** | 05218 | 71 | 8 | 11.3 | 0.0251 |
| **Platelet activation** | 04611 | 127 | 12 | 9.4 | 0.0255 |
| **Bile secretion** | 04976 | 72 | 8 | 11.1 | 0.0270 |
| **Regulation of lipolysis in adipocytes** | 04923 | 59 | 7 | 11.9 | 0.0272 |
| **Focal adhesion** | 04510 | 206 | 17 | 8.3 | 0.0300 |
| **Wnt signalling pathway** | 04310 | 145 | 13 | 9.0 | 0.0301 |
| **Transcriptional misregulation in cancer** | 05202 | 176 | 15 | 8.5 | 0.0312 |
| **Adherens junction** | 04520 | 74 | 8 | 10.8 | 0.0312 |
| **Adrenergic signalling in cardiomyocytes** | 04261 | 148 | 13 | 8.8 | 0.0348 |
| **MAPK signalling pathway** | 04010 | 259 | 20 | 7.7 | 0.0366 |
| **cAMP signalling pathway** | 04024 | 196 | 16 | 8.2 | 0.0375 |
| **Protein processing in endoplasmic reticulum** | 04141 | 165 | 14 | 8.5 | 0.0376 |
| **Leukocyte transendothelial migration** | 04670 | 120 | 11 | 9.2 | 0.0381 |
| **Dilated cardiomyopathy** | 05414 | 91 | 9 | 9.9 | 0.0382 |
| **Protein export** | 03060 | 26 | 4 | 15.4 | 0.0387 |
| **RIG-I-like receptor signalling pathway** | 04622 | 64 | 7 | 10.9 | 0.0401 |
| **FoxO signalling pathway** | 04068 | 136 | 12 | 8.8 | 0.0402 |
| **Chagas disease (American trypanosomiasis)** | 05142 | 107 | 10 | 9.3 | 0.0417 |
| **Type II diabetes mellitus** | 04930 | 52 | 6 | 11.5 | 0.0444 |
| **Hepatitis B** | 05161 | 139 | 12 | 8.6 | 0.0462 |
| **TNF signalling pathway** | 04668 | 110 | 10 | 9.1 | 0.0488 |
| **Bacterial invasion of epithelial cells** | 05100 | 81 | 8 | 9.9 | 0.0493 |

**Supplementary Table 4.** Pathways significantly (*p*<0.05) enriched with DEGs (*p*<0.001) after comparing complete denervated animals (Tx) with Sham-denervated animals (Sham). *Pathway rno* indicates the pathway number for *rattus norvegicus;* *n genes* the number of genes in the pathway; o*verlap* the number of genes of the pathway present in list of DEGs; *% affected* the overlap between the genes in the pathway and the genes in list of DEGs expressed as a percentage; *p-value* the level of significance in enrichment.

| pathway | pathway rno | n genes | overlap | % affected | *p*-value |
| --- | --- | --- | --- | --- | --- |
| **FoxO signalling pathway** | 04068 | 136 | 22 | 16.2 | 0.0000 |
| **N-Glycan biosynthesis** | 00510 | 50 | 11 | 22.0 | 0.0000 |
| **Protein processing in endoplasmic reticulum** | 04141 | 165 | 22 | 13.3 | 0.0000 |
| **Non-alcoholic fatty liver disease (NAFLD)** | 04932 | 163 | 18 | 11 | 0.0014 |
| **Ubiquitin mediated proteolysis** | 04120 | 141 | 16 | 11.3 | 0.0019 |
| **Complement and coagulation cascades** | 04610 | 83 | 11 | 13.3 | 0.0028 |
| **Circadian rhythm** | 04710 | 30 | 6 | 20.0 | 0.0033 |
| **Insulin resistance** | 04931 | 111 | 13 | 11.7 | 0.0037 |
| **Metabolic pathways** | 01100 | 1308 | 87 | 6.7 | 0.0042 |
| **Chronic myeloid leukaemia** | 05220 | 76 | 10 | 13.2 | 0.0045 |
| **Longevity regulating pathway - multiple species** | 04213 | 65 | 9 | 13.8 | 0.0049 |
| **Signalling pathways regulating pluripotency of stem cells** | 04550 | 142 | 15 | 10.6 | 0.0051 |
| **Acute myeloid leukaemia** | 05221 | 57 | 8 | 14.0 | 0.0072 |
| **Insulin signalling pathway** | 04910 | 140 | 14 | 10.0 | 0.0106 |
| **Adherens junction** | 04520 | 74 | 9 | 12.2 | 0.0113 |
| **Adipocytokine signalling pathway** | 04920 | 75 | 9 | 21.0 | 0.0123 |
| **AMPK signalling pathway** | 04152 | 129 | 13 | 10.1 | 0.0126 |
| **Thyroid cancer** | 05216 | 29 | 5 | 17.2 | 0.0135 |
| **AGE-RAGE signalling pathway in diabetic complications** | 04933 | 104 | 11 | 10.6 | 0.0149 |
| **Starch and sucrose metabolism** | 00500 | 53 | 7 | 13.2 | 0.0159 |
| **Pyrimidine metabolism** | 00240 | 107 | 11 | 10.3 | 0.0181 |
| **Glycosaminoglycan degradation** | 00531 | 21 | 4 | 19.0 | 0.0188 |
| **Galactose metabolism** | 00052 | 32 | 5 | 15.6 | 0.0203 |
| **RNA polymerase** | 03020 | 32 | 5 | 15.6 | 0.0203 |
| **Non-small cell lung cancer** | 05223 | 56 | 7 | 12.5 | 0.0210 |
| **Cell cycle** | 04110 | 127 | 12 | 9.4 | 0.0255 |
| **Terpenoid backbone biosynthesis** | 00900 | 23 | 4 | 17.4 | 0.0258 |
| **Cysteine and methionine metabolism** | 00270 | 47 | 6 | 12.8 | 0.0288 |
| **Proteasome** | 03050 | 47 | 6 | 12.8 | 0.0288 |
| **Fatty acid biosynthesis** | 00061 | 14 | 3 | 21.4 | 0.0300 |
| **mTOR signalling pathway** | 04150 | 160 | 14 | 8.8 | 0.0301 |
| **Prolactin signalling pathway** | 04917 | 76 | 8 | 10.5 | 0.0358 |
| **Glycosphingolipid biosynthesis - ganglio series** | 00604 | 15 | 3 | 20.0 | 0.0362 |
| **Thyroid hormone signalling pathway** | 04919 | 119 | 11 | 9.2 | 0.0362 |
| **ErbB signalling pathway** | 04012 | 91 | 9 | 9.9 | 0.0382 |
| **Protein export** | 03060 | 26 | 4 | 15.4 | 0.0387 |
| **Jak-STAT signalling pathway** | 04630 | 151 | 13 | 8.6 | 0.0399 |
| **Colorectal cancer** | 05210 | 64 | 7 | 10.9 | 0.0401 |
| **Glycosaminoglycan biosynthesis - keratan sulfate** | 00533 | 16 | 3 | 18.8 | 0.0429 |
| **Maturity onset diabetes of the young** | 04950 | 27 | 4 | 14.8 | 0.0437 |
| **Arginine and proline metabolism** | 00330 | 52 | 6 | 11.5 | 0.0444 |
| **Endometrial cancer** | 05213 | 52 | 6 | 11.5 | 0.0444 |
| **Longevity regulating pathway** | 04211 | 94 | 9 | 9.6 | 0.0455 |

**Supplementary Table 5.** Pathways significantly (*p*<0.05) enriched with DEGs (*p*<0.001) after comparing Sham-DARK and Sham-LAN animals. *Pathway rno* indicates the pathway number for *rattus norvegicus;* *n genes* the number of genes in the pathway; o*verlap* the number of genes of the pathway present in list of DEGs; *% affected* the overlap between the genes in the pathway and the genes in list of DEGs expressed as a percentage; *p-value* the level of significance in enrichment.

| Pathway | pathway rno | n genes | overlap | % affected | *p*-value |
| --- | --- | --- | --- | --- | --- |
| **Oxytocin signalling pathway** | 04921 | 160 | 21 | 13.1 | 0.0001 |
| **Oestrogen signalling pathway** | 04915 | 96 | 15 | 15.6 | 0.0001 |
| **Influenza A** | 05164 | 171 | 21 | 12.3 | 0.0001 |
| **Adherens junction** | 04520 | 74 | 12 | 16.2 | 0.0003 |
| **Gap junction** | 04540 | 88 | 13 | 14.7 | 0.0004 |
| **Thyroid hormone signalling pathway** | 04919 | 119 | 15 | 12.6 | 0.0009 |
| **Oocyte meiosis** | 04114 | 113 | 14 | 12.3 | 0.0015 |
| **Toxoplasmosis** | 05145 | 123 | 14 | 11.3 | 0.0034 |
| **Cell cycle** | 04110 | 127 | 14 | 11.0 | 0.0046 |
| **Amphetamine addiction** | 05031 | 65 | 9 | 13.8 | 0.0049 |
| **Glucagon signalling pathway** | 04922 | 102 | 12 | 11.7 | 0.0049 |
| **Epstein-Barr virus infection** | 05169 | 231 | 21 | 9.1 | 0.0062 |
| **Prion diseases** | 05020 | 34 | 6 | 17.6 | 0.0062 |
| **Phosphatidylinositol signalling system** | 04070 | 96 | 11 | 11.4 | 0.0085 |
| **Vascular smooth muscle contraction** | 04270 | 123 | 13 | 10.5 | 0.0087 |
| **Circadian entrainment** | 04713 | 97 | 11 | 11.3 | 0.0091 |
| **Platelet activation** | 04611 | 127 | 13 | 10.2 | 0.0112 |
| **Apoptosis** | 04210 | 141 | 14 | 9.9 | 0.0112 |
| **Salivary secretion** | 04970 | 76 | 9 | 11.8 | 0.0134 |
| **Dopaminergic synapse** | 04728 | 130 | 13 | 10.0 | 0.0134 |
| **Glycine, serine and threonine metabolism** | 00260 | 40 | 6 | 15.0 | 0.0138 |
| **Longevity regulating pathway - multiple species** | 04213 | 65 | 8 | 12.3 | 0.0154 |
| **GnRH signalling pathway** | 04912 | 92 | 10 | 10.9 | 0.0166 |
| **Osteoclast differentiation** | 04380 | 134 | 13 | 9.7 | 0.0169 |
| **Renin secretion** | 04924 | 67 | 8 | 11.9 | 0.0183 |
| **Aldosterone synthesis and secretion** | 04925 | 84 | 9 | 10.7 | 0.0243 |
| **Bile secretion** | 04976 | 72 | 8 | 11.1 | 0.0270 |
| **Pertussis** | 05133 | 73 | 8 | 10.9 | 0.0290 |
| **Glutamatergic synapse** | 04724 | 115 | 11 | 9.5 | 0.0292 |
| **Inflammatory mediator regulation of TRP channels** | 04750 | 115 | 11 | 9.7 | 0.0292 |
| **Pathways in cancer** | 05200 | 400 | 29 | 7.2 | 0.0298 |
| **Inositol phosphate metabolism** | 00562 | 74 | 8 | 10.8 | 0.0312 |
| **Prostate cancer** | 05215 | 89 | 9 | 10.1 | 0.0338 |
| **Adrenergic signalling in cardiomyocytes** | 04261 | 148 | 13 | 8.7 | 0.0348 |
| **Progesterone-mediated oocyte maturation** | 04914 | 90 | 9 | 10.0 | 0.0359 |
| **Leukocyte transendothelial migration** | 04670 | 120 | 11 | 9.1 | 0.0381 |
| **Fc gamma R-mediated phagocytosis** | 04666 | 91 | 9 | 9.1 | 0.0382 |
| **Amoebiasis** | 05146 | 106 | 10 | 9.4 | 0.0395 |
| **Morphine addiction** | 05032 | 92 | 9 | 9.7 | 0.0405 |
| **Rheumatoid arthritis** | 05323 | 92 | 9 | 9.7 | 0.0405 |
| **Chagas disease (American trypanosomiasis)** | 05142 | 107 | 10 | 9.3 | 0.0417 |
| **Glycosaminoglycan biosynthesis - keratan sulfate** | 00533 | 16 | 3 | 18.7 | 0.0429 |
| **Long-term potentiation** | 04720 | 65 | 7 | 10.7 | 0.0431 |
| **Central carbon metabolism in cancer** | 05230 | 65 | 7 | 10.7 | 0.0431 |
| **Notch signalling pathway** | 04330 | 52 | 6 | 11.5 | 0.0444 |
| **African trypanosomiasis** | 05143 | 40 | 5 | 12.5 | 0.0479 |
| **Homologous recombination** | 03440 | 28 | 4 | 14.2 | 0.0490 |

**Supplementary Table 6.** Common pathways significantly (*p*<0.05) enriched by DEGs (*p*<0.001) in both Sham-LAN and Tx-LAN when compared to Sham-DARK. Sorted on significance in Sham-LAN. *Pathway rno* indicates the pathway number for *rattus norvegicus;* *n genes* the number of genes in the pathway; o*verlap* the number of genes of the pathway present in list of DEGs; *% affected* the overlap between the genes in the pathway and the genes in list of DEGs expressed as a percentage; *p-value* the level of significance in enrichment.

| Pathway name | Pathway nr | n genes | overlap in Sham-LAN | % affected | *p*-value | overlap in Tx-LAN | % affected | *p*-value |
| --- | --- | --- | --- | --- | --- | --- | --- | --- |
| **Influenza A** | 05164 | 171 | 21 | 12.3 | 0.0001 | 18 | 10.5 | 0.0024 |
| **Thyroid hormone signalling pathway** | 04919 | 119 | 15 | 12.6 | 0.0009 | 12 | 10.1 | 0.0161 |
| **Epstein-Barr virus infection** | 05169 | 231 | 21 | 9.1 | 0.0062 | 21 | 9.1 | 0.0062 |
| **Phosphatidylinositol signalling system** | 04070 | 96 | 11 | 11.5 | 0.0085 | 11 | 11.5 | 0.0085 |
| **Apoptosis** | 04210 | 141 | 14 | 9.9 | 0.0112 | 14 | 9.9 | 0.0112 |
| **Glycine, serine and threonine metabolism** | 00260 | 40 | 6 | 15.0 | 0.0138 | 7 | 17.5 | 0.0034 |
| **Bile secretion** | 04976 | 72 | 8 | 11.1 | 0.0270 | 12 | 16.7 | 0.0002 |
| **Pathways in cancer** | 05200 | 400 | 29 | 7.3 | 0.0298 | 31 | 7.8 | 0.0110 |
| **Inositol phosphate metabolism** | 00562 | 74 | 8 | 10.8 | 0.0312 | 8 | 10.8 | 0.0312 |
| **Adrenergic signalling in cardiomyocytes** | 04261 | 148 | 13 | 8.8 | 0.0348 | 14 | 9.5 | 0.0166 |
| **Leukocyte transendothelial migration** | 04670 | 120 | 11 | 9.2 | 0.0381 | 11 | 9.2 | 0.0381 |
| **Fc gamma R-mediated phagocytosis** | 04666 | 91 | 9 | 9.9 | 0.0382 | 9 | 9.9 | 0.0382 |
| **Amoebiasis** | 05146 | 106 | 10 | 9.4 | 0.0395 | 11 | 10.4 | 0.0170 |
| **Central carbon metabolism in cancer** | 05230 | 65 | 7 | 10.8 | 0.0431 | 7 | 10.8 | 0.0431 |
